# Comparison of Two Methods for Correcting Baseline Offset Error in Phase-Contrast MR Imaging

**DOI:** 10.1186/1532-429X-18-S1-P334

**Published:** 2016-01-27

**Authors:** Yang Lin, Ke Jiang, Yiu-Cho Chung

**Affiliations:** 1 Shenzhen Institutes of Advanced Technology, Chinese Academy of Sciences, Shenzhen, China; 2 grid.59053.3a0000000121679639University of Science and Technology of China, Hefei, China

## Background

Phase-contrast MRI (PC-MRI) can be used to assess valvular heart diseases. It can also measure pulmonary-systemic flow ratio (Qp/Qs) and help identify intracardiac shunts. However, phase offsets in PC-MRI caused by main field inhomogeneity and eddy-current introduce baseline offset errors in flow quantification and hence Qp/Qs. This error can be corrected by surface-fitting [1] or using a separate phantom acquisition [2]. A recent study found that the phantom acquisition method did not help reduce error of Qp/Qs [3]. This study compared the effectiveness of the two baseline correction methods in reducing phase errors when measuring Qp/Qs in healthy volunteers using PC-MRI.

## Methods

Ten healthy volunteers were recruited for this IRB approved study. Each volunteer gave informed consent. The study was performed on a 3.0 T MRI clinical scanner (Trio Tim, Siemens, Germany). In each subject, localizers were used to find the aortic and pulmonary outflow tract. Velocity encoded, retrogated gradient echo cine was used to measure blood flow perpendicular to the two outflow tracts. Imaging parameters were: TR/TE = 4.4, flip angle = 20°, slice thickness = 5.5 mm, matrix size = 192 × 144, bandwidth = 704 Hz/pixel, VENC = 170 cm/s, 5 lines per heartbeat, 20 reconstructed phases. After flow measurements, a water phantom was put inside the scanner. It was scanned with identical flow imaging protocols after waiting for five minutes [4]. Baseline correction using phantom was performed following [3]. Baseline correction by the surface-fitting method was performed using Qflow (Medis, Netherland). Qp/Qs was calculated for each volunteer.

## Results

**Figure 1:**
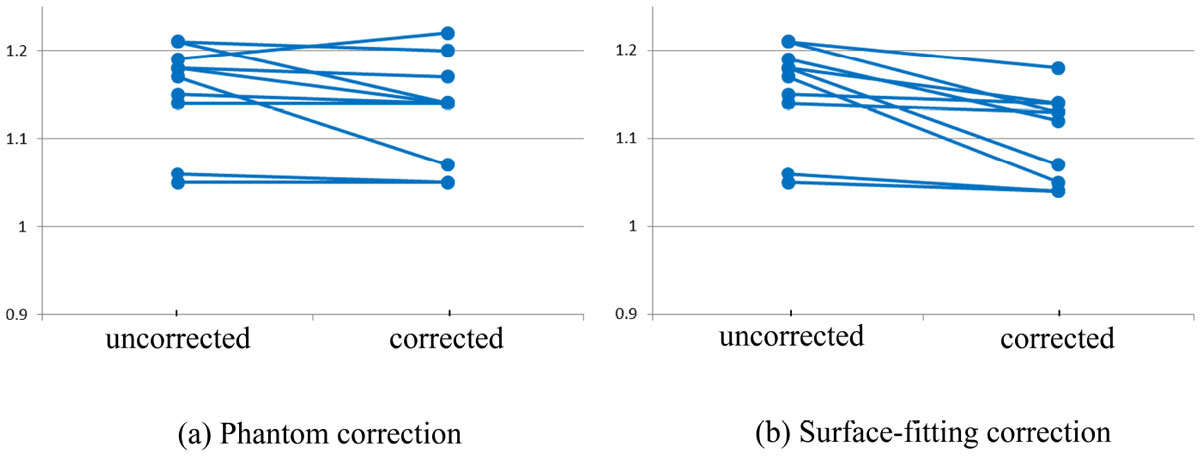
**Qp/Qs ratio of the healthy volunteers before and after baseline correction. (a) Correction using phantom; (b) correction using surface-fitting**.

Table 1 lists the mean and standard deviation of Qp/Qs ratios before and after correction using the two methods. The surface-fitting method brought the Qp/Qs ratio closer to 1 than the phantom method. Figure 1 shows how the two correction methods changed the Qp/Qs ratio in individual cases. Qp/Qs ratios were lower (p < 0.05) after being corrected by the surface-fitting method. Reduction of Qp/Qs ratios using the phantom correction method was not statistically significant (p = 0.1 ).

**Table 1:** Summary of aortic flow, pulmonic flow and Qp/Qs before and after baseline correction

	Aortic flow (mL)	Pulmonic flow (mL)	Qp/Qs
Before correction	86.0 ± 15.1	99.6 ± 19.6	1.16 ± 0.06
After phantom correction	86.5 ± 14.7	98.1 ± 19.1	1.13 ± 0.06
After surface-fitting correction	87.1 ± 15.4	96.2 ± 18.6	1.10 ± 0.05

## Conclusions

The surface-fitting method reduced the Qp/Qs ratios in all cases while the phantom correction method increased the ratio in one case. As the phantom correction method needs additional scanning time, the surface-fitting approach would be preferred for baseline offset correction in PC-MRI.
